# *Ex Situ* Left Ventricular Pressure-Volume Loop Analyses for Donor Hearts: Proof of Concept in an Ovine Experimental Model

**DOI:** 10.3389/ti.2024.12982

**Published:** 2024-07-11

**Authors:** I. A. Ertugrul, R. A. D. A. Puspitarani, B. Wijntjes, M. T. Vervoorn, E. M. Ballan, N. P. van der Kaaij, H. van Goor, B. D. Westenbrink, A. van der Plaats, F. Nijhuis, V. van Suylen, M. E. Erasmus

**Affiliations:** ^1^ Department of Cardiothoracic Surgery, University Medical Centre Groningen, University of Groningen, Groningen, Netherlands; ^2^ Department of Cardiology, University Medical Centre Groningen, University of Groningen, Groningen, Netherlands; ^3^ XVIVO, Gothenburg, Sweden; ^4^ Department of Cardiothoracic Surgery, University Medical Centre Utrecht, Utrecht, Netherlands; ^5^ Department of Cardiology, Laboratory of Experimental Cardiology, University Medical Center Utrecht, Utrecht, Netherlands; ^6^ Netherlands Heart Institute, Utrecht, Netherlands; ^7^ Department of Medical Biology and Pathology, University Medical Centre Groningen, University of Groningen, Groningen, Netherlands

**Keywords:** donation after circulatory death (DCD), *ex situ* heart perfusion, viability assessment, functional evaluation, heart transplantation

## Abstract

*Ex situ* heart perfusion (ESHP) has emerged as an important strategy to preserve donation after brain death (DBD) and donation after circulatory death (DCD) donor hearts. Clinically, both DBD and DCD hearts are successfully preserved using ESHP. Viability assessment is currently based on biochemical values, while a reliable method for graft function assessment in a physiologic working mode is unavailable. As functional assessment during ESHP has demonstrated the highest predictive value of outcome post-transplantation, this is an important area for improvement. In this study, a novel method for *ex situ* assessment of left ventricular function with pressure-volume loop analyses is evaluated. Ovine hearts were functionally evaluated during normothermic ESHP with the novel pressure-volume loop system. This system provides an afterload and adjustable preload to the left ventricle. By increasing the preload and measuring end-systolic elastance, the system could successfully assess the left ventricular function. End-systolic elastance at 60 min and 120 min was 2.8 ± 1.8 mmHg/mL and 2.7 ± 0.7 mmHg/mL, respectively. In this study we show a novel method for functional graft assessment with *ex situ* pressure-loop analyses during ESHP. When further validated, this method for pressure-volume assessments, could be used for better graft selection in both DBD and DCD donor hearts.

## Graphical Abstract

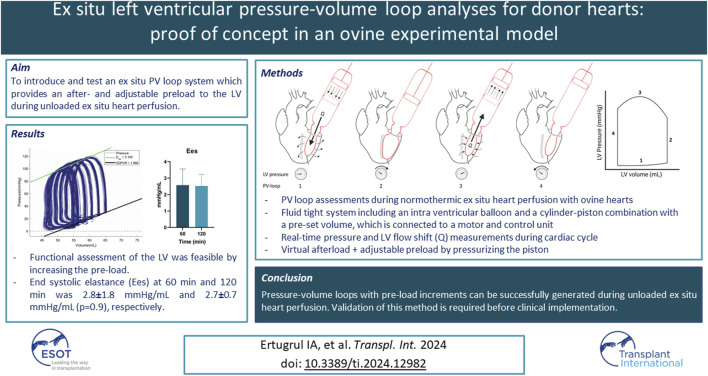

## Introduction

*Ex situ* heart perfusion (ESHP) has been successfully implemented in the field of donor heart preservation worldwide [1, 2]. ESHP prevents ischemia during preservation by providing oxygenated, nutrient-rich perfusate to the heart and provides a platform for graft assessment prior to heart transplantation (HTx) [1]. In donation after brain death (DBD), both hypothermic and normothermic ESHP have been shown to be non-inferior to the static cold storage and have been successfully applied for extended criteria DBD donors [3–5]. In addition, ESHP has emerged as a successful preservation technique in donation after circulatory death (DCD). In DCD heart donation, hearts are retrieved from donors whose death is determined by circulatory arrest, as opposed to the golden standard DBD. Transplantation of DCD hearts has expanded the donor pool and number of heart transplantations substantially, thereby offering a viable solution for the persistent shortage of donor hearts [6]. However, the utilization of DCD hearts presents challenges, given the inherent ischemic injury and thus the need for optimized preservation techniques, including ESHP [6, 7].

The Transmedics Organ Care System™ (Andover, MA, United States) is currently the only clinically available normothermic ESHP device and provides coronary perfusion without loading the ventricles [8]. Viability can only be assessed by measuring lactate levels, and the platform does not offer a module for the assessment of myocardial function. Lactate was previously shown to be a poor predictor of outcome after transplantation [9]. In addition, an increasing number of studies indicate that evaluating donor heart viability with functional ventricle loaded parameters is superior compared to metabolic parameters. This suggests that the assessment of cardiac contractile function could emerge as a reliable tool for graft evaluation and decision-making in HTx [10–13]. However, there is currently no clinically available device capable for *ex situ* assessment of contractile function of the heart including clinically important factors such as pre- and afterload.

In this study we describe our initial experience with a novel device that enables functional graft assessment during unloaded normothermic ESHP with pressure-volume (PV) loop analyses, in an ovine abattoir model. This device provides virtual afterload and preload during ESHP in contrast to the non-compressible classical Langendorff balloon. Using the ovine abattoir model, we aimed to develop a widely available and reproducible model, recapitulating the DCD setting.

## Materials and Methods

### Animals

Female sheep (10 months old, 30–35 kg) from the abattoir were included in this study. The protocol is consistent with regulation number 1069/2009 of the European Parliament and Council regarding abattoir animal products for diagnosis and research, which were authorized by the relevant legal animal welfare authorities (Food and Consumer Product Safety Authority). The sheep were terminated employing a captive bolt pistol to the head. Subsequently, the carotid arteries were cut open and the animal was suspended by its hind legs to exsanguinate. During exsanguination, blood was collected and heparinized (5.000 IU/L). A median sternotomy was performed, and the heart and lungs were harvested *en bloc*.

### Heart Procurement

On the back table, the heart was immediately topically cooled and visually inspected for abnormalities or damage due to the termination process. The heart was dissected from the lungs by transecting the pulmonary artery and veins. Approximately 10 cm of the ascending aorta was retained and canulated by securing the cardioplegia flush line in the aperture of the ascending aorta. Therefore, both coronary sinuses were perfused simultaneously. The cardioplegic solution (Custodiol histidine-tryptophan-ketoglutarate solution, Essential Pharmaceuticals, LLC, Durham, United States) was supplemented with adenosine (200 μmol/L), lidocaine (500 μmol/L) and nitroglycerin (100 mg/L) and administered at a pressure of 80–100 mmHg (1L, 4°C) [14, 15]. Warm ischemic time was defined as the time between the termination of the animal and delivery of cardioplegia. A maximum of 10 min of warm ischemia was accepted.

### Subnormothermic *Ex Situ* Heart Perfusion

All hearts were preserved with oxygenated subnormothermic ESHP (15°C) for 2 h. The subnormothermic ESHP system (modified Kidney Assist Transport, XVIVO, Gothenburg, Sweden) consists of a reservoir, an oxygenator (Medos Hilite 1000 XENIOS, Heilbronn, Germany), and a centrifugal pump (Medos DP2 XENIOS, Heilbronn, Germany). Antegrade perfusion of the coronary arteries was delivered in a pressure-controlled and pulsatile fashion by cannulating the aorta root. The aortic valve was tested for insufficiency. Oxygen (100%) was delivered at a flow of 100 mL/min. Hearts were continuously perfused with a hyperkalemic (20 mmol/L), buffered dextran-albumin perfusion solution supplemented with nitroglycerin (25 mg/L). Perfusion temperature was targeted at 15°C. The hearts were prepared for subnormothermic ESHP by cannulating the aorta and by venting the LV to avoid LV distention. Subsequently, the hearts were submerged within the reservoir and the perfusion cannula was attached to the arterial end of the perfusion system. The perfusion pressure was gradually increased to 45 mmHg, aiming at a perfusion flow of >150 mL/min. The right atrium was left open, enabling the free return of the perfusate from the coronary sinus into the reservoir. Blood gases and electrolytes were analyzed to ensure the perfusate composition (pH: 7.1–7.4; HCO_3_^−^: 20–25 mmol/L; pO_2_: 40–60 kPa; pCO_2_: 4.7–6 kPa; K^+^: >20 mmol/L; Ca^2+^: 0.18–0.25 mmol/L).

### Normothermic *Ex Situ* Heart Perfusion

At the end of preservation, the hearts were submerged in cold cardioplegic solution in order to minimize injury during the preparations for normothermic evaluation. First, hearts were flushed with Perfadex (XVIVO, Gothenburg, Sweden) to flush out the hyperkalemic subnormothermic ESHP perfusate. Subsequently, hearts were weighted. For normothermic evaluation, a Langendorff perfusion system comparable to Langendorff perfusion was used. The normothermic ESHP system (modified Kidney Assist, XVIVO, Gothenburg, Sweden) consists of a heater-cooler and a pump housing unit, which must be connected to a disposable set, consisting of a reservoir, an oxygenator (Medos Hilite 2800 XENIOS, Heilbronn, Germany) and a centrifugal pump (Medos DP2 XENIOS, Heilbronn, Germany). The normothermic perfusion is pressure-controlled and delivers a continuous flow to the coronary arteries using the same cannula as for subnormothermic ESHP. Initial reperfusion was initiated in a controlled way, with a gradual increase in both pressure and temperature [16]. Perfusion temperature was initiated at 27°C with 3°C increments every 5 min over 30 min. After 30 min, the pressure was increased from 40 mmHg to 60 mmHg. Rewarming continued for another 30 min. The perfusate was based on albumin solution supplemented with Perfadex (XVIVO, Gothenburg, Sweden), to which washed RBCs (±500 mL) were added. The gas flow of carbogen (O_2_ 95%; CO_2_ 5%) was adjusted to maintain physiological pH and arterial carbon dioxide pressure (paCO_2_). Blood gasses and electrolytes are analyzed to ensure a perfusate composition within the physiological range (pH: 7.3–7.4; HCO_3_^−^: 20–25 mmol/L; pO_2_: 40–60 kPa; pCO_2_: 4.7–6 kPa; K^+^: 3.0–5.9 mmol/L; Ca^2+^: 1.0–1.3 mmol/L; Hb: 4.0–4.5 g/dL). Reperfusion was initiated with a low calcium concentration (<0.8 mmol/L). During normothermic *ex situ* perfusion, hearts were ventricular paced at a rate of 70 beats/min. Normothermic ESHP was performed for a total duration of 2 h.

### *Ex situ* Pressure Volume Loop Analyses

**FIGURE 1:**
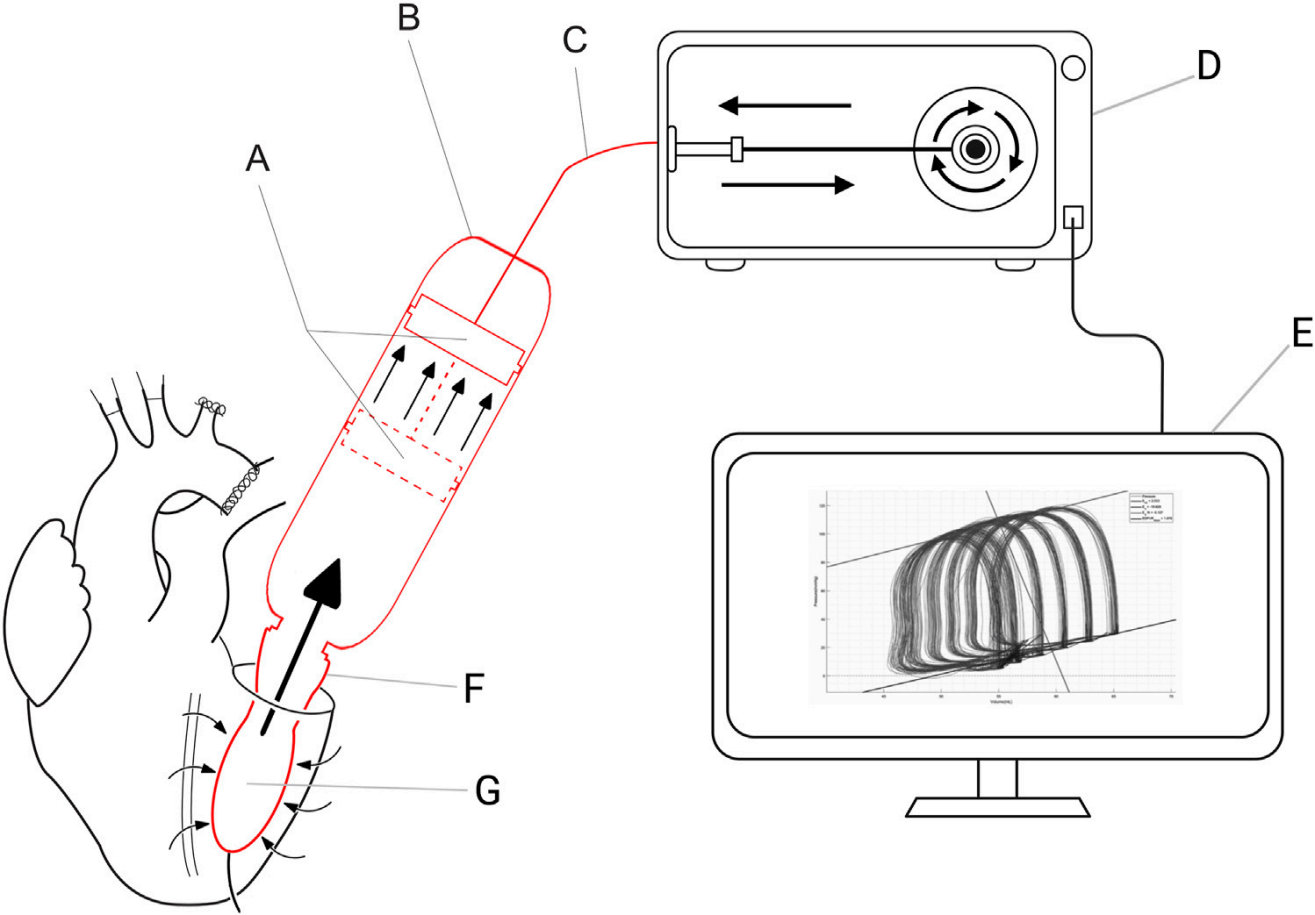
XVIVO pressure-volume loop analysis platform. **(A)**: piston, **(B)**: cylinder, **(C)**: Bowden cable, **(D)**: motor generating force for pressurizing the volume within the cylinder, **(E)**: control unit and data output, **(F)**: mitral valve cannula, **(G)**: intra-ventricular balloon.

The novel XVIVO *ex situ* PV loop system consists of a cylinder-piston combination that is placed on the annulus of the mitral valve, a balloon, a motor, and a control unit (Figure 1). The balloon is placed in the LV through the mitral valve, secured with a transapical line, and filled with saline. As stated before, the LV is vented. This assures that there will be little to no fluid surrounding the intra-ventricular balloon. To make sure that the balloon does not protrude from the LV to the left atrium, the mitral valve annulus is closed by placing two polyamide nylon tapes (Ethicon 3 mm × 70) behind the chordae tendineae of both the posterior and anterior leaflet of the mitral valve. Subsequently, the nylon bands are secured using a tourniquet kit, which ensures that the mitral valve annulus completely encloses the mitral valve cannula. A WheatStone bridge pressure catheter is placed in the balloon for real-time pressure measurements. The balloon is connected to the mitral valve cannula, which forms the connection to the piston-cylinder combination (Figures 1A, B). As a result, the volume within the balloon is in continuity with the volume within the cylinder-piston combination, providing a fluid-tight system with a constant and preset fluid volume. As the LV contracts, the volume is pushed out of the balloon into the cylinder, moving the piston (Figure 1A). This movement of the piston is transmitted through a Bowden cable (Figure 1C) which is connected to the motor pulley (Figure 1D), registering the volume changes in the LV during the cardiac cycle. Both the motor and pressure sensor are connected to the control unit which includes a computer with XVIVO software (Figure 1E).

**FIGURE 2:**
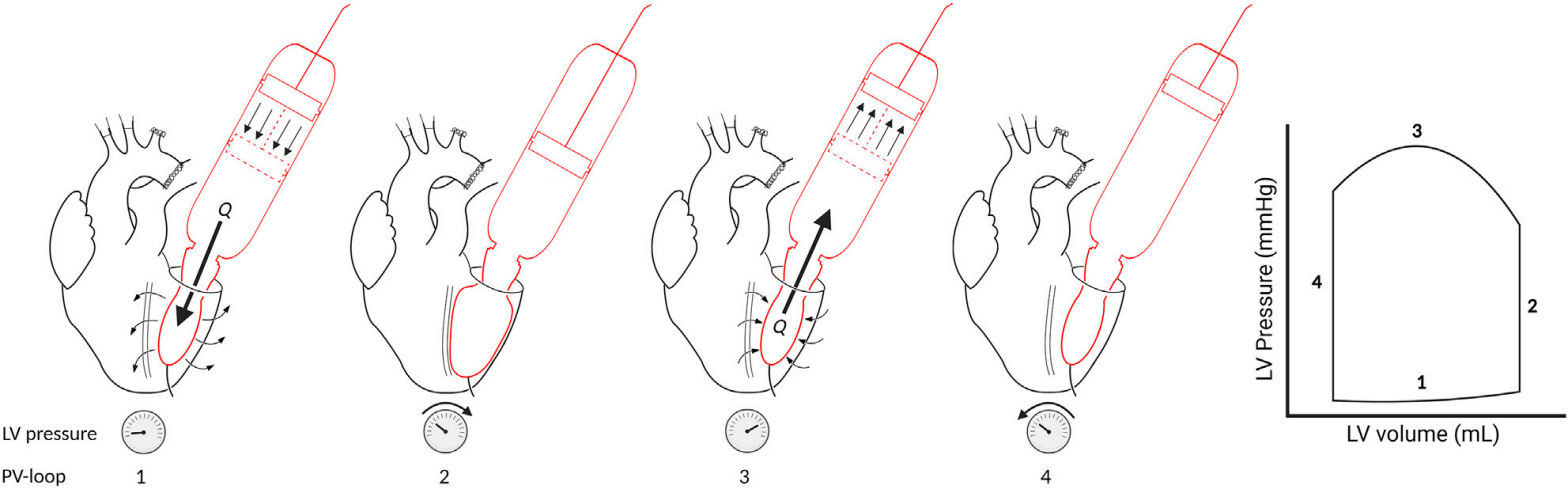
Four phases of the cardiac cycle in PV loop. Phase 1: Diastolic filling. The intra-ventricular balloon will be filled therefore Q [flow (mL/min)] is in the direction of the LV and pressure will slightly rise. Phase 2: Isovolumetric contraction. The LV pressure will rise until the threshold pressure is reached. Phase 3: Ejection. The intra-ventricular balloon will be emptied (Q is in the direction of the cylinder). Phase 4: Isovolumetric relaxation. LV pressure will fall.

During the cardiac cycle the software switches between the four phases of the cardiac cycle (Figure 2). The software detects the changes in the cardiac cycle by an algorithm dedicated to every phase of the cardiac cycle. Change in the cardiac phase is detected by the software as follows. The software switches from the diastolic filling phase to the isovolumetric contraction phase when a small outward volume displacement from the ventricular cavity is detected. It switches from the isovolumetric contraction phase to the systolic ejection phase when a certain predetermined threshold pressure is reached. The systolic ejection phase changes to the isovolumetric relaxation phase when a small inward volume displacement to the ventricular cavity is detected. And lastly, the software switches from the isovolumetric relaxation phase to the diastolic filling phase when the ventricular pressure is lower than the predetermined preload pressure.

Furthermore, the volume within the cylinder-piston combination is pressurized by force generated by the motor. Therefore, the internal volume can be pressurized by changing the motor power. This enables the simulation of a virtual physiologic load on the ventricle (pre- and afterload). Both the power and speed delivered by the motor can be controlled with the XVIVO software. The software reads the pressure measured in the LV, compares this with the desired preload pressure, and subsequently increases or decreases the motor power to adjust the LV pressure. The desired preload pressure can be adjusted in the XVIVO software after every cardiac cycle. As a result, the LV pressure will increase to the desired preload pressure within one cardiac cycle, enabling the ability to effortlessly execute a preload challenge to assess LV function.

**FIGURE 3:**
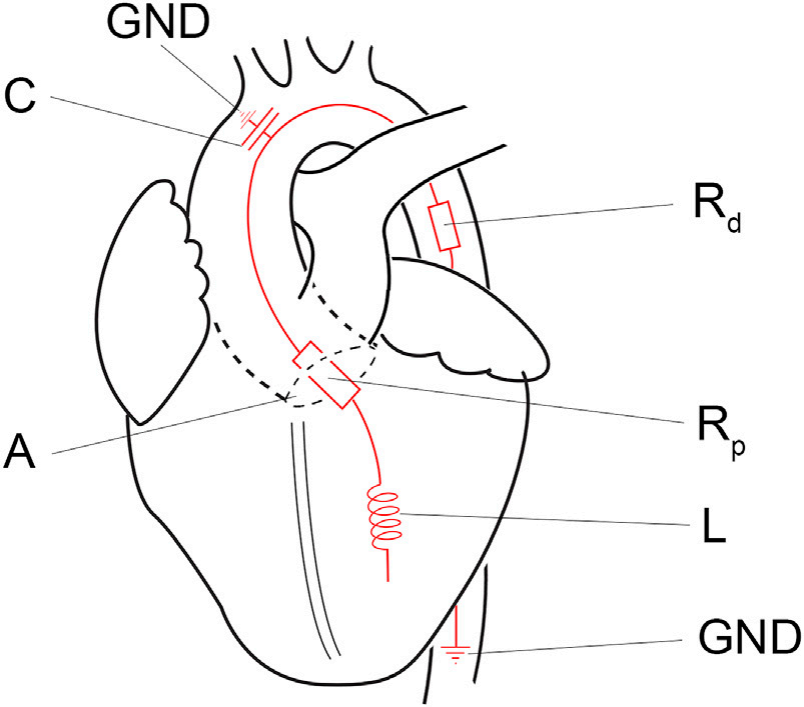
Simplified electronic equivalent fluidic impedance of the human body. Conducted from Kung et al. [17]. A, aortic valve; C, compliance component of the fluiding impedance; GND, ground; L, the inertia component, Rd, distal resistance; Rp, proximal resistance.

In the systolic ejection phase, the software controls the afterload. The afterload is calculated from the measured ventricular outward flow and a virtual impedance based on the analogue described by Kung and colleagues [17]. The simplified virtual impedance is constructed out of four components (Figure 3; Table 1). The inertia component (L) counteracts a flow change. Compliance (C), primarily the aortic compliance, is the stretching capacity of the arteries. R_p_ is the virtual flow resistance of the aortic valve, and R_d_ is the virtual flow resistance of the distal track/the systemic vascular resistance (SVR).

**TABLE 1:** Components of virtual impedance.

Components of virtual impedance	Symbol	Standard setting
Inertia component	L (Barye s^2^/cm^3^)	0.7
Inverse compliance	1/C	420
Flow resistance aortic valve	R_p_ (Barye s/cm^3^)	24.5
Flow resistance distal track/SVR	R_d_ (Barye s/cm^3^)	404.6

The afterload is calculated using the following equation:

$$P=L\frac{dQ}{dt}+Q\;\cdot \;R_{p}+\frac{1}{C}\int \left(Q-Q_{Rd}\right)dt+P_{C_{0}}$$

In which P is the target pressure in mmHg. The software will control the motor force in such a way that the measured pressure in the ventricle will equal this targeted pressure or afterload. Q is the flow (mL/min) out of the ventricle and Q_Rd_ is the flow through the virtual flow resistance of the SVR. The inertia component (L) is dependent on the flow change (dQ) over time (dt, in seconds) out of the ventricle. R_p_ is the flow resistance of the aortic valve. Pressure generated by the compliance component (C) is determent by the volume in the aorta. Volume in the aorta can be calculated by summation of the flow from the ventricle (Q), minus the flow to the body (Qrd). P_c0_ is the pressure (in mmHg) in the aorta at the start of the systolic ejection phase. The targeted pressure of the ventricular cavity is recalculated at intervals of dt, and dt is defined as to be smaller than 4 milliseconds.

The equation above is valid in the systolic ejection phase, as in the other phases the aortic valve would be closed. If the aortic valve is closed, the aorta is still loaded and stretched as a result of the systolic ejection phase. During unloading of the aorta, the aortic pressure falls. The formula for decreasing the pressure of the aorta, is based on a resistance-capacitor circuit, the electronic equivalent of resistance and compliance:

$$P_{c}=P_{ces}\;\cdot \;e^{-\frac{t}{R_{d}}\;\times \;C\;}$$

In which P_c_ is the virtual pressure in the aorta, P_ces_ is the end systolic pressure compliance in the aorta and t is the progression in time since closure of the aortic valve in seconds, C is the compliance and R_d_ is the SVR. When a heartbeat is in the isovolumetric systolic phase, P_c_ is the threshold pressure for switching to the ejection phase.

### Data Collection

#### Blood Gas Analysis

Blood gas analysis was performed with the ABL90 Flex plus blood gas analyzer (Radiometer Benelux BV). Analyses were performed during subnormothermic ESHP (5, 60 and 120 min), after priming of the normothermic ESHP system and during normothermic ESHP (5, 30, 60, 90 and 120 min).

#### PV Loop Analysis

At 60 and 120 min of normothermic ESHP, PV loop analyses were performed. Before testing, the pacing was haltered, and hearts were checked for their rhythm. During PV loop analyses, pre-load was started at 10 mmHg and increased to 25 mmHg in steps of 5 mmHg. After each increment of 5 mmHg, PV loops were recorded for 60 s. Calculations and figures were made in MATLAB (The Mathworks).

### Statistical Analysis

Data is presented as mean ± standard deviation (SD). A repeated measures ANOVA was performed to analyze differences over time. *p*-value <0.05 was considered statistically significant. Data analysis was performed using GraphPad Prism 9.4 (GraphPad Software, CA, United States).

## Results

### Baseline Data, Ischemic and Preservation Time

A total of 5 female sheep of 10–12 months old (25–35 kg) were included in the study. The functional warm ischemic time was 8.4 ± 0.9 min. The cold ischemic time during preparations for subnormothermic ESHP was 5 ± 0.5 min. Hearts were perfused with subnormothermic ESHP for 135 ± 13.3 min and evaluated during normothermic ESHP for 122 ± 1.6 min. The cold ischemic time during preparations for normothermic ESHP was 9 ± 3.4 min. The baseline weight of the heart was 328.4 ± 3 g. Weight gain during preservation was 71 ± 12.7 g (*p* = 0.0005) and during normothermic ESHP 41 ± 16 g (*p* = 0.010).

### Subnormothermic Machine Perfusion

**FIGURE 4:**
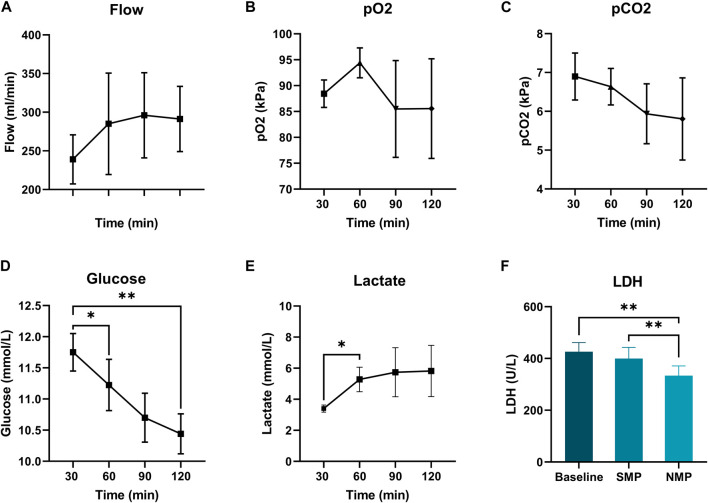
Subnormothermic machine perfusion. **(A)**: Coronary flow (mL/min), **(B)**: Arterial pCO_2_ (kPa), **(D)**: Glucose levels (mmol/L), **(C)**: Arterial pCO_2_ (kPa), **(E)**: Lactate levels (mmol/L), **(F)**: Lactate dehydrogenase (U/L).

All hearts were perfused with a pressure of 45 mmHg. Coronary flow increased non-significantly over time until 60 min of preservation, and stabilized hereafter (Figure 4A). paO_2_ and paCO_2_ concentration during subnormothermic ESHP are shown in Figures 4B, C, respectively. Glucose concentration decreased over the course of preservation (Figure 4D), baseline: 11.8 ± 0.3, end of subnormothermic ESHP: 10.4 ± 0.3 (*p* = 0.005). A significant increase in lactate was seen in the first hour of preservation (Figure 4E). However, after 60 min of preservation, lactate remained rather stable. Lactate dehydrogenase after 5 min of preservation was 426 ± 35 U/L (Figure 4F). At the end of preservation, lactate dehydrogenase decreased to 400 ± 43 U/L (*p* = 0.3).

### Normothermic Machine Perfusion

**FIGURE 5:**
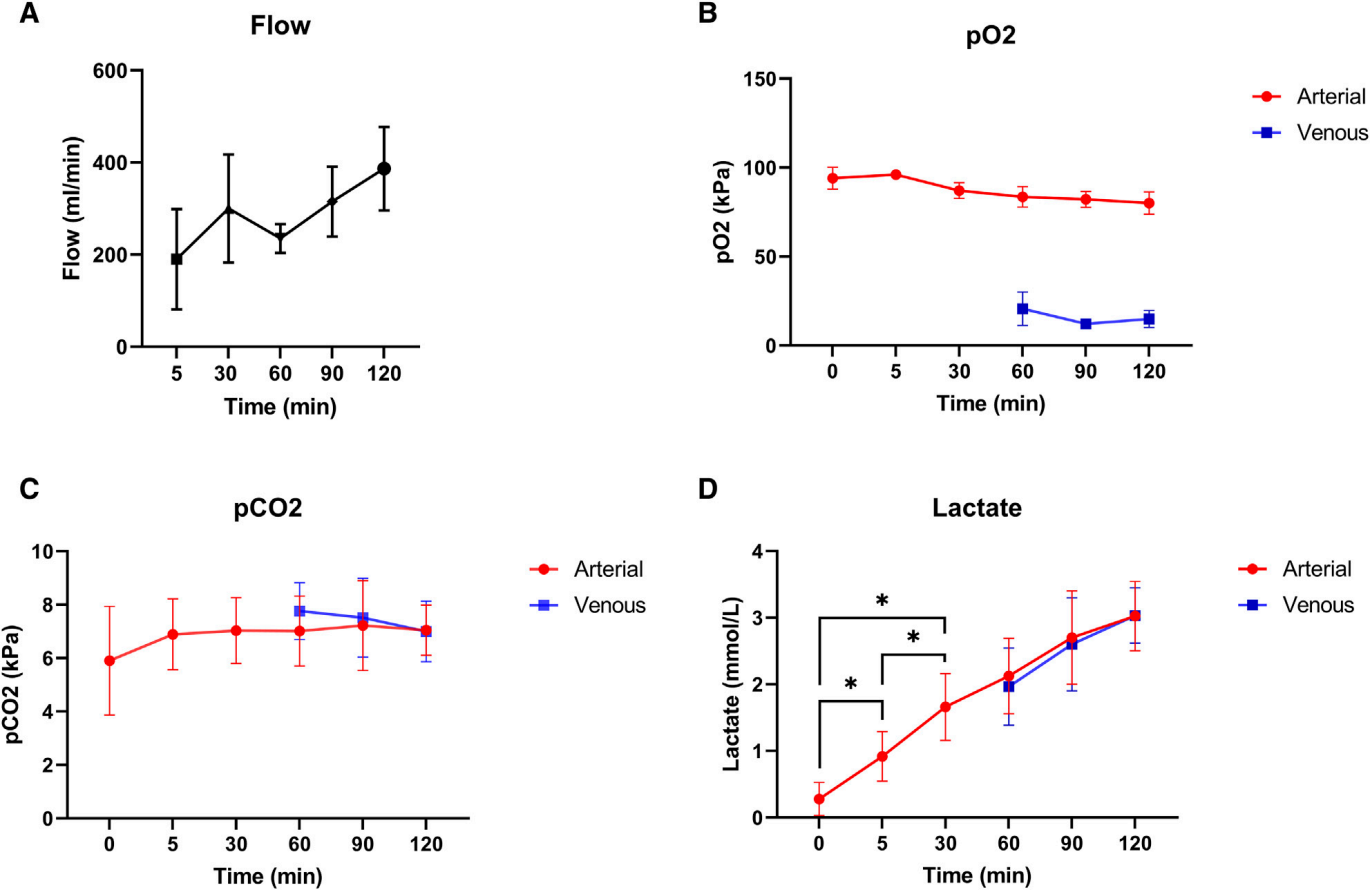
Normothermic machine perfusion. **(A)**: Coronary flow (mL/min), **(B)**: pO_2_ (kPa), **(C)**: pCO_2_ (kPa), **(D)**: Lactate levels (mmol/L).

Mean pressure during normothermic ESHP was 62 ± 6 mmHg. Changes in flow during normothermic ESHP, are shown in Figure 5A. Changes in both arterial and venous pO_2_ and pCO_2_ are shown in Figures 5B, C. In the first 30 min of normothermic ESHP, lactate increased significantly (baseline: 0.28 mmol/L, 5 min: 0.92 mmol/L, 30 min: 1.66 mmol/L, *p* = 0.02). Hereafter, a non-significant increase in lactate was seen between each timepoint (Figure 5D). At the end of normothermic ESHP, lactate dehydrogenase level was 333 ± 37 U/L (*p* = 0.005, Figure 4D).

### PV Loop Analyses

**FIGURE 6:**
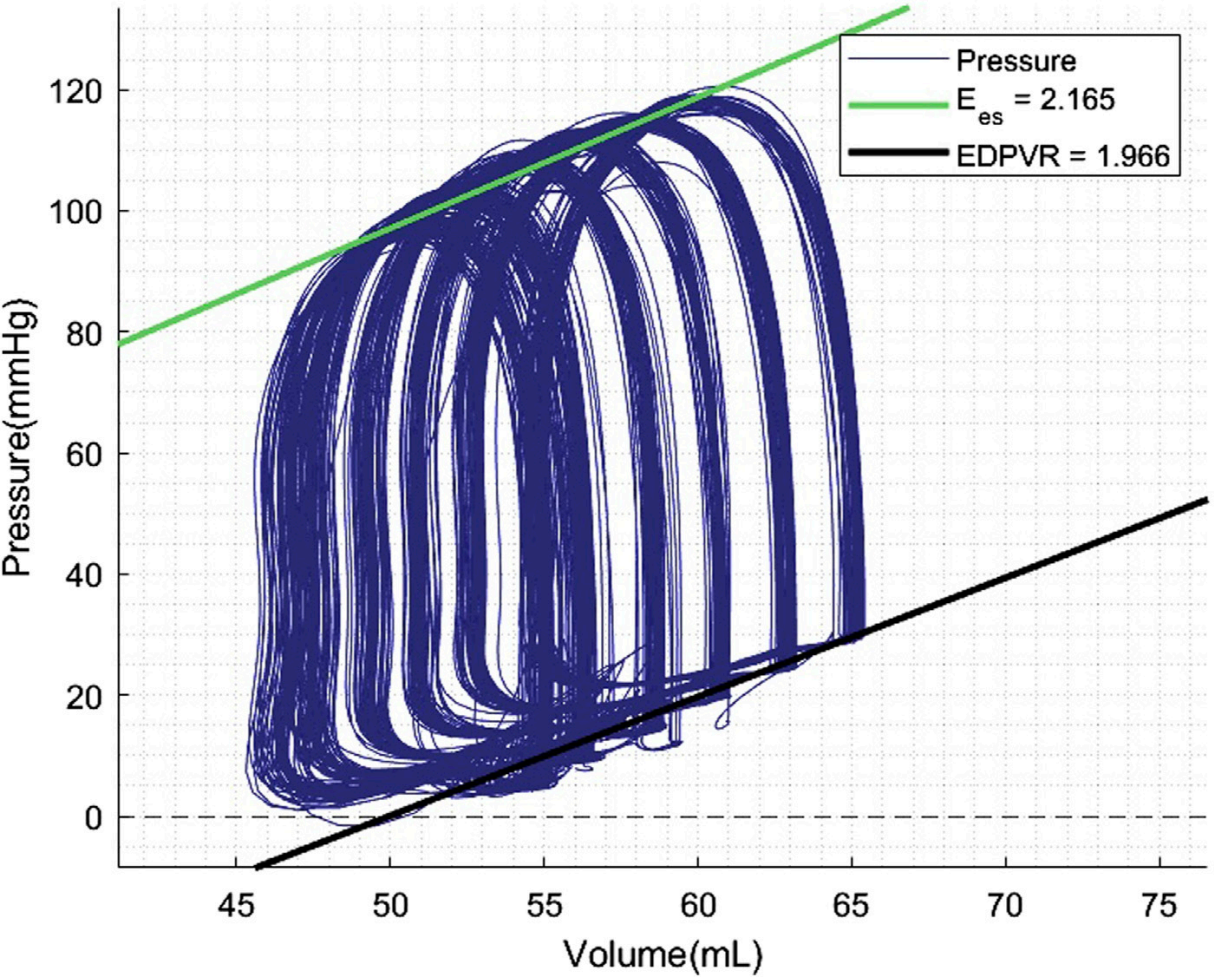
Pressure-volume loop analyses output. Preload was increased with increments of 5 mmHg. EDPVR, End-diastolic pressure-volume relationship; Ees, End-systolic elastance.

**FIGURE 7:**
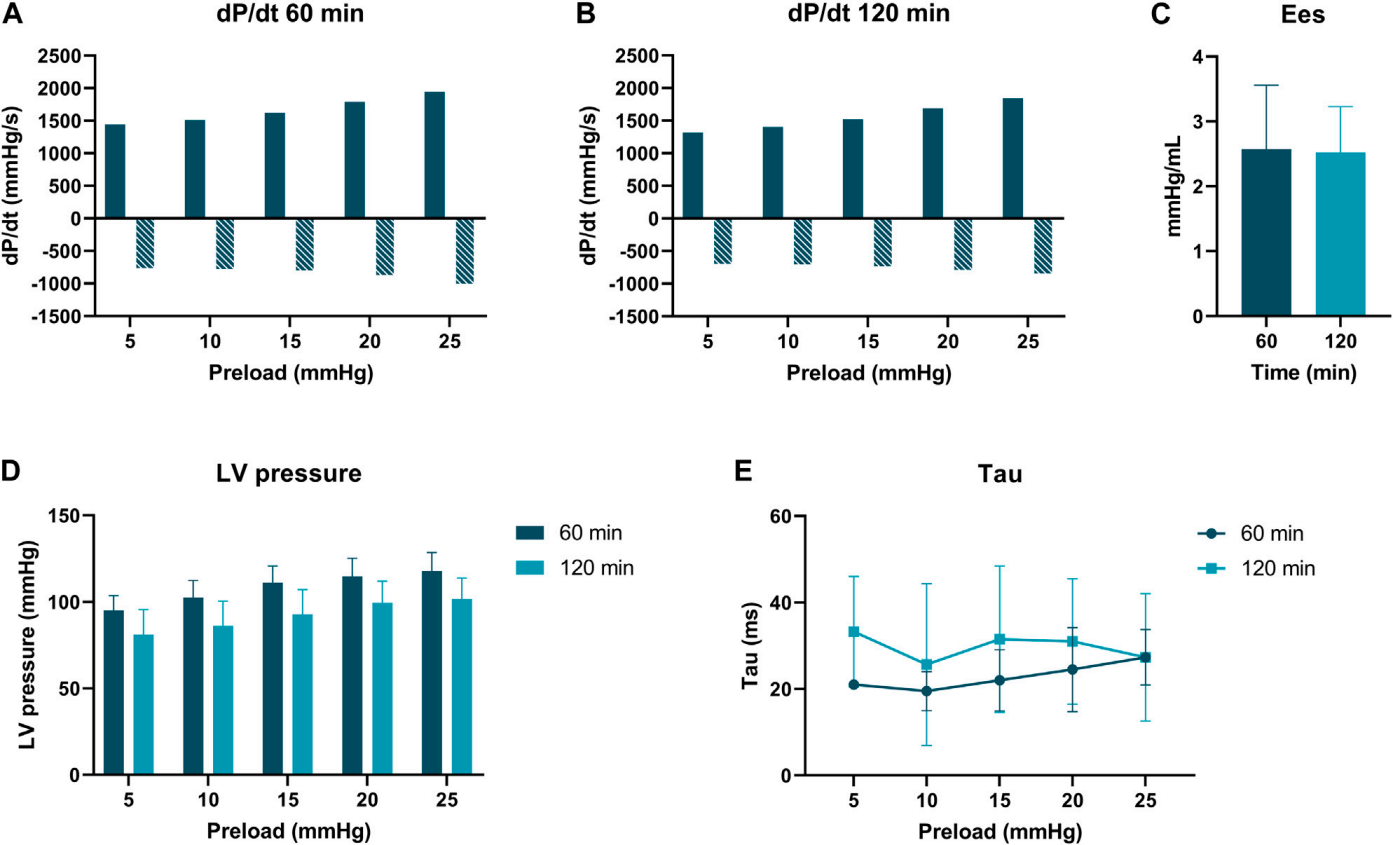
Pressure-volume loop analyses. **(A)** dP/dt min and max (mmHg/s) at 60 min of *ex situ* heart perfusion. **(B)**: dP/dt min and max (mmHg/s) at 120 min of *ex situ* heart perfusion **(C)**: End-systolic elastance (mmHg/mL), **(D)**: LV pressure (mmHg) **(E)**: Tau (ms).

PV loop analyses were performed at 60 and 120 min of normothermic ESHP. A simulated output from one of the PV loop assessments with increasing pre-load is shown in Figure 6. Dp/dt-min and -max and maximally generated pressure values are shown in Figure 7. End-systolic elastance (Ees) at 60 min and 120 min of normothermic ESHP was 2.8 ± 1.8 mmHg/mL and 2.7 ± 0.7 mmHg/mL (*p* = 0.9), respectively (Figure 7C). Tau, the exponential decay of the ventricular pressure during isovolumic relaxation, is shown in Figure 7E.

## Discussion

In this study, a novel method for *ex situ* PV loop analyses during unloaded normothermic ESHP is shown in an ovine abattoir model, which is recapitulating the DCD setting. This PV loop system provides a virtual afterload and an adjustable preload to the LV. Pre-load increments of 5 mmHg, led to measurable PV loops for LV function assessment. Our results, including end-systolic elastance, are in line with experimental studies on LV function in ovine models [19–21].

Given the uncertainty about transiency and reversibility of myocardial dysfunction in DCD hearts, assessment of cardiac function might be of added value. In line with this, it is important to state that several donor hearts initially thought to be transplantable were discarded after perfusion on the Transmedics OCS system [1]. Furthermore, in DBD HTx, DBD donors might suffer from brain stem death-induced myocardial dysfunction, which could prove to be reversible over time [22]. Prolonging the interval between brain death and graft assessment, may therefore increase the number of successful heart procurements, which stresses the transiency of myocardial dysfunctions over time, especially from extended criteria DBD donors [22, 23]. Therefore, donor heart assessment in both DCD and DBD could be of additional value.

Several methods have been applied to assess graft function during ESHP. Echocardiography is the preferred imaging technique for myocardial contractility. Two-dimensional echocardiography has been used during ESHP to determine myocardial contraction by assessing fractional area reduction [24]. Lowalekar et al. showed that cardiac functional parameters and ventricular and septal wall thicknesses can be assessed with a two-dimensional transesophageal probe during ESHP [25]. Furthermore, more extensive methods including the quantitative assessment of shear wave velocity using ultrasound have been used as a biomarker for cardiac viability in a porcine model. This methodology includes ultrasound elastography and assessment of myocardial stiffness evolution during ESHP. Shear wave velocity has been shown to correlate with cardiac function index, suggesting shear wave velocity as a non-invasive marker of graft viability [26].

PV loop analyses are widely used in basic research and are considered the gold standard for *in situ* hemodynamic assessment of the heart [27]. PV loop assessments have also been applied during normothermic regional perfusion in DCD HTx to assess the donor heart before retrieval [12]. Messer et al. demonstrated the effectiveness of this functional assessment method for DCD HTx with successful transplantation and 100% survival rate [28]. PV loop analyses are also performed during EHSP using an intraventricular conductance catheter and isovolumetric pressure measurements [12, 18, 29].

The novel *ex situ* PV loop system includes a pre-determined virtual afterload and an adjustable preload, and therefore simulates a physiological loading state, which is a valuable novel addition to ESHP PV loop assessments. Contrary to current PV loop methods, this novel system allows LV fluid shuttling. In addition, previous studies have shown that ESHP in working mode may result in improved preservation compared to non-working or unloaded perfusion [30]. This novel *ex situ* PV loop analyses platform provides an accessible working mode ESHP system during Langendorff perfusion and may therefore optimize ESHP. In addition, complicated working-mode or loaded perfusion systems can be avoided. Furthermore, its compact and transportable size, easy application and modest design might facilitate clinical application.

For optimal graft viability assessment, we believe that a multi-assessment approach including real-time PV loop assessment with our novel system, myocardial perfusion and metabolic- and damage markers would be an adequate strategy for evaluation of contractility, coronary flow, and overall cardiac function. Several metabolic markers, including myocardial oxygen consumption, lactate, glucose, and metabolomics have been applied in ESHP graft assessment [31–34]. These markers are accessible and could provide additional insight in graft viability.

Several experimental rodent models of cardiac DCD are available, providing a relatively easily manageable model with respect to technical and financial aspects [28, 35, 36]. However, large animal models provide a more clinically relevant platform for DCD heart research. Although these models are clinically relevant, costs are relatively high and challenging to perform. Utilizing animal organs obtained from an abattoir is an alternative source of animal donor hearts and fits well into the 3R’s principle (refinement, reduction, and replacement) of humane experimental techniques. Since these animals are specifically bred and terminated for consumption, repurposing their organs for research after termination allows for scientific research without causing additional animal harm [37]. Other advantages of abattoir models include low costs and unlimited access. In large-animal models, the porcine heart is one of the preferred choices considering its anatomical similarity to the human heart [38, 39]. However, ovine hearts are also comparable on a physiological and anatomical level to human heart and have been studied comprehensively [40–42]. The advantage of using ovine hearts for experimental studies is that the slaughter process for sheep is shorter than that for pigs. Therefore, acceptable warm ischemic times can be reached. The described ovine model is a reproducible model for ESHP DCD studies.

Further enhancements of the PV loop system could include the possibility to adjust the afterload and assessment of right-ventricular function. As this is a proof-of-concept study, we did not include a cut-off value for adequate cardiac function. Future studies validating this novel *ex situ* PV loop system are required to determine a cut-off value relevant for clinical DCD HTx. In addition, these studies should test the safety of the intra-ventricular balloon. The intra-ventricular balloon and mitral valve canula affects the geometry of the mitral valve and might therefore also affect the geometry of the LV, which can be considered as a limiting factor of this study. Further limiting factors include the low number of hearts and lack of a control group (e.g., laboratory-raised sheep). However, this paper aimed at showing a novel animal DCD model and system for *ex situ* PV loop analyses as a proof of concept, not to validate these methods. Although hearts retrieved from an abattoir have many advantages, there are also limitations to abattoir organs for research. Variability in farms of origin, stress due to transport and sacrifice, and a high-fat diet, are some of the factors which should be considered when using abattoir models for experimental studies [39].

In conclusion, this study shows a reproducible method for DCD heart preservation and proofs the concept of functional assessment of these ovine hearts. We tested a novel clinically applicable method for functional assessment of the graft with *ex situ* PV loop analyses including a virtual afterload and preload during ESHP. We believe that this method could become important in more optimal graft selection and enhancing overall outcome in both DBD and DCD HTx. Validation of this system is the next step in the development of this method and is required before clinical implementation.

## Data Availability

The raw data supporting the conclusions of this article will be made available by the authors, without undue reservation.

## Abbreviations

DBD, Donation after brain death; DCD, Donation after circulatory death; Ees, End systolic elastance; ESHP, *ex situ* heart perfusion; HTx, Heart transplantation; LV, Left ventricle; PV, Pressure-volume; SVR, Systemic vascular resistance.
